# Semiconductivity induced by spin–orbit coupling in Pb_9_Cu(PO_4_)_6_O

**DOI:** 10.1038/s41598-023-48383-z

**Published:** 2023-11-30

**Authors:** Hua Bai, Jianrong Ye, Lei Gao, Chunhua Zeng, Wuming Liu

**Affiliations:** 1 Institute of Physical and Engineering Science/Faculty of Science, Kunming University of Science and Technology, Kunming, 650500 China; 2 Beijing National Laboratory for Condensed Matter Physics, Institute of Physics, Chinese Academy of Sciences, Beijing, 100190 China

**Keywords:** Materials science, Physics

## Abstract

Recently, a possible room-temperature superconductor known as LK-99 (Pb_10-x_Cu_x_(PO_4_)_6_O (0.9 < x < 1.1)) has sparked a wave of research. However, many experimental works have proven that it is a semiconductor. At the same time, many theoretical works have reached the conclusion that it is a flat band metal. The inconsistency between theoretical and experimental works may be caused by neglecting the spin–orbit coupling effect in calculations. We performed calculations of electronic structure of Pb_9_Cu(PO_4_)_6_O with spin–orbit coupling, and the results show that it's indeed a semiconductor, not a metal. In the ferromagnetic state it is an indirect-bandgap semiconductor with a bandgap of 292 meV. While in the antiferromagnetic-A state, it is a direct-bandgap semiconductor with a bandgap of 300 meV. Our work provides a possible explanation for the contradictions of previous experiments and theories, and provides some theoretical basis for the potential application of Pb_9_Cu(PO_4_)_6_O as a semiconductor.

## Introduction

Recently, a group from South Korea reported a possible room-temperature and atmospheric-pressure superconductor LK-99, which has attracted great attention worldwide^1^. Room-temperature and atmospheric-pressure superconductor is a very desirable goal of human beings. Although many high-temperature superconducting systems have been discovered^2–4^, superconductors at room-temperature and atmospheric- pressure have never been discovered yet. If the superconductivity of LK-99 is confirmed, it will be a huge achievement.

As reported, LK-99 is a compound that replaces part of Pb with Cu in lead apatite, and its chemical formula is Pb_10-x_Cu_x_(PO_4_)_6_O (0.9 < x < 1.1)^1^. After this report, large amounts of experimental works were carried out on a global scale to try to reproduce LK-99 and study its properties^5–13^. It is worth noting that many experiments have determined that LK-99 is a semiconductor^6,9–13^. At the same time, a large number of theoretical results have also been reported^14–23^, and most assume that LK-99 is a metal with two flat bands at the Fermi level, this is not consistent with the above experimental results. In fact, all previous first-principles calculations have not considered the spin–orbit coupling (SOC) effect. However, in systems with heavy elements, the SOC effect is often relatively strong^24^. And there are many heavy elements Pb in LK-99, so its SOC effect should not be ignored. This may be the reason why the above calculations and experiments are inconsistent.

In this work, we used first-principles method to calculate in detail the crystal structure and electronic structure of LK-99 in the case of x = 1 with the chemical formula of Pb_9_Cu(PO_4_)_6_O. First, we identified the most stable structure and a metastable structure. Then, for the most stable structure, we calculated its electronic structure without and with considering the SOC. If the SOC is ignored, our calculations agree with most previous first-principles calculations^14–20^. However, the calculations considering SOC show that Pb_9_Cu(PO_4_)_6_O is not a metal but a semiconductor. In the ferromagnetic (FM) state, SOC will cause the band inversion of two flat bands and open a bandgap, making Pb_9_Cu(PO_4_)_6_O an indirect-bandgap semiconductor, which is consistent with some current experimental results^6,9–13^. Its magnetic moment is about 1 μB and its easy-axis is *c*-axis. In addition, a small amount of electronic doping makes Pb_9_Cu(PO_4_)_6_O metallic with a very narrow flat band at the Fermi level. While in the antiferromagnetic-A (AFM-A) state, Pb_9_Cu(PO_4_)_6_O is a direct-bandgap semiconductor.

## Results and discussions

### Structures of Pb_10_(PO_4_)_6_O and LK-99

**Figure 1:**
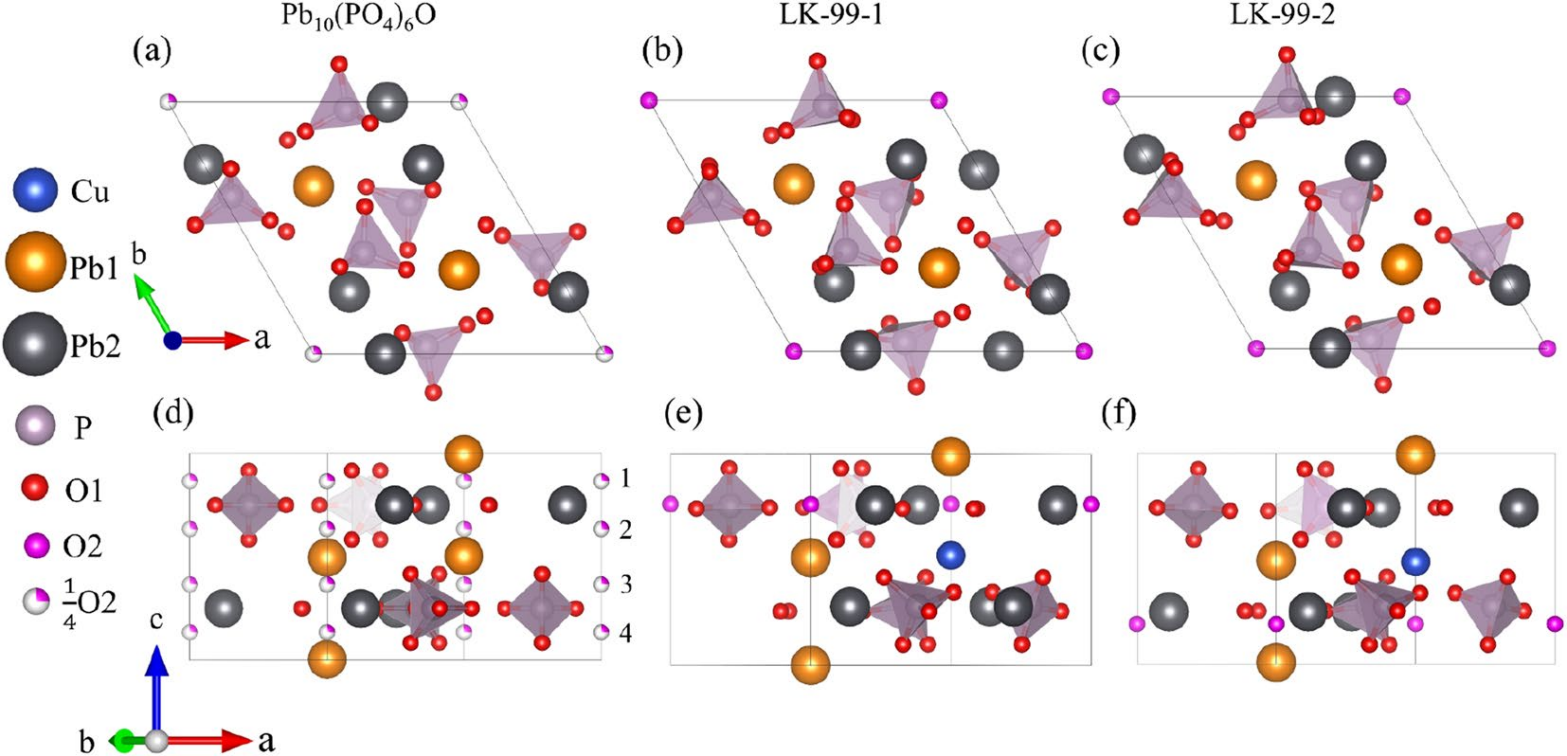
Crystal structures of Pb_10_(PO_4_)_6_O, LK-99–1 and LK-99–2. (**a**–**c**) Top view. (**d**–**f**) Side view. In the structure of Pb_10_(PO_4_)_6_O, the O2 atoms are 1/4 occupied in the 4 positions shown in (**d**).

Figure 1a and d show the crystal structure of Pb_10_(PO_4_)_6_O, which is the parent compound of LK-99. It has a hexagonal structure with space group 176 (*P*6_3_/*m*). In Pb_10_(PO_4_)_6_O, Pb atoms have two symmetrically equivalent positions: Pb1 and Pb2, as shown in the Fig. 1. A unit cell contains four Pb1 atoms and six Pb2 atoms. The O atoms also have two symmetrically equivalent positions: O1 and O2. The twenty-four O1 atoms and six P atoms form six triangular cone-shaped PO_4_ units, while the O2 atoms form a one-dimensional chain along the *c*-direction. It should be noted here that O2 atoms are all 1/4 occupied in the four fully equivalent positions. Previous first-principles calculation work has shown that Pb_10_(PO_4_)_6_O is a semiconductor^15^, and previous experimental work has shown that the chemical formula of LK-99 is Pb_10-x_Cu_x_(PO_4_)_6_O (0.9 < x < 1.1). In addition, previous theoretical work has demonstrated that the structure in which Pb1 atoms are occupied is more stable than the structure in which Pb2 atoms are occupied^16^. For convenience, we consider the case of x = 1. Since the positions of the four Pb1 atoms are completely equivalent, we first replaced one of them with Cu atoms. At this time, due to the change in symmetry, the positions of the four O2 atoms become unequal. In order to find the most stable O2 atom position, we choose one position to place an O2 atom each time, remove the other three O2 atoms, and then optimize the structure. In the end we only get two structures. If we put an O2 atom in position 1 or 2 as shown in Fig. 1d, it will become the same structure after optimization, as shown in Fig. 1e. Similarly, placing an O2 atom at the position 3 or 4 and then optimizing it also results in another same structure, as shown in Fig. 1f. This result shows that, in Pb_9_Cu_1_(PO_4_)_6_O, only two O2 atom sites are stable. In order to distinguish the two structures, we named the former as LK-99-1 and the latter as LK-99-2. Both LK-99-1 and LK-99-2 have a trigonal crystal structure with space group 143 (*P*3). In addition, compared with the parent compound, in LK-99-1 and LK-99-2, the position of Pb atoms and the PO_4_ units away from Cu atom does not change much, but the three PO_4_ units near the Cu atom all undergo obvious rotation. Furthermore, the total energy of LK-99–2 is lower than that of LK-99–1 in the FM and NM states by 0.26 eV and 0.21 eV, respectively. That is, LK-99-2 is the most stable structure, while LK-99-1 is a metastable structure.

### Electronic structure without SOC

**Figure 2:**
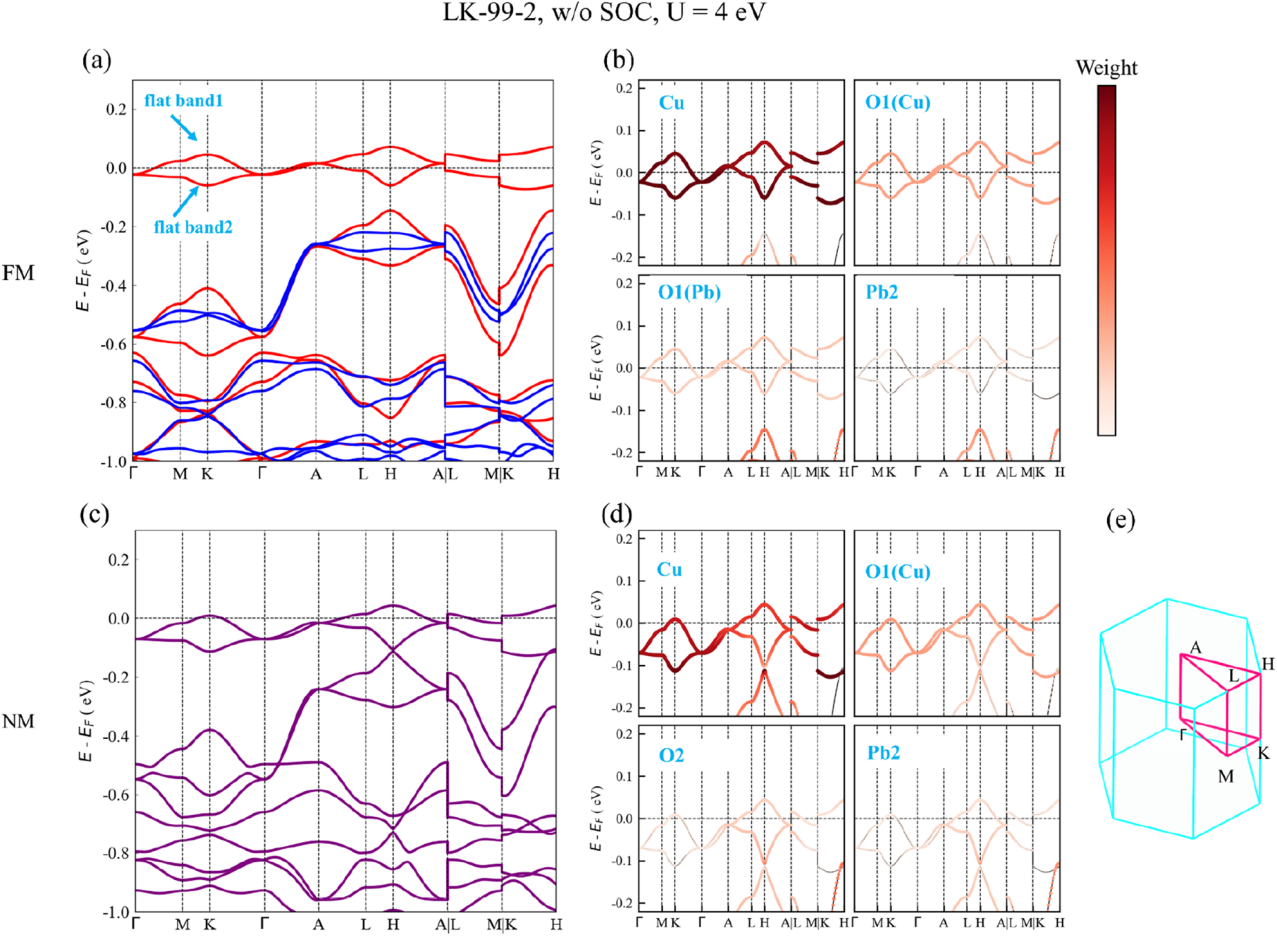
(**a**,**c**) Band structures of LK-99-2 without SOC (w/o SOC), and the Hubbard interaction U = 4 eV. (**a**) Ferromagnetic (FM) state, where the red and dark blue lines represent spin-up bands and spin-down bands, respectively. The blue arrows point to flat band1 and flat band2. (**c**) Non-magnetic (NM) state. (**b**,**d**) Corresponding projected band structures around flat bands in FM state and NM state, respectively. The darker the color, the greater the contribution of the selected element. O1(Cu) and O1(Pb) represent O1 atoms near Cu and near Pb, respectively. (**e**) Brillouin zone and high symmetry points, and paths for band calculations.

Some previous works have calculated the electronic structure of LK-99 without considering the SOC, and found two flat bands at the Fermi level^14–16^. To carefully verify these results, we first performed electronic state calculations without considering the SOC. Figures 2 and S1 show the band structures of LK-99-2 and LK-99-1 without SOC for both ferromagnetic (FM) and non-magnetic (NM) states, and the Hubbard interaction U = 4 eV. The total energy of the FM state is 0.15 and 0.20 eV lower than that of the NM state in LK-99-1 and LK-99-2, respectively. After Cu substitution, both LK-99-1 and LK-99-2 become metals in the FM and the NM states. What's more interesting is that there are two flat bands near the Fermi level of these four band structures. We call the upper and lower flat bands as flat band1 and flat band2 respectively, as shown in Fig. 2a. Now that LK-99-2 is more stable, we will focus on it next. The flat band1 and flat band2 have some different characteristics: the highest energy point and the lowest energy point of flat band1 are at H point and Γ point, respectively, while those of flat band2 are at A point and the middle of K-H line. In the FM state, both flat bands are partially filled and fully spin polarized, while in the NM state, flat band1 is partially filled but flat band2 is fully occupied. In order to analyze the composition of states near the Fermi level, the corresponding projected band structures are also calculated, as shown in Fig. 2b,d and Fig. S1b,d. In the NM state of LK-99-1 and LK-99-2, the states near the Fermi level are mainly contributed by Cu. In addition, O2, O1 around Cu also have partial contribution. While in the FM state of both, the states near the Fermi level are also mainly contributed by Cu, moreover, O1 and Pb2 also have partial contribution. Overall, spin polarization causes a slight change in the composition of states near the Fermi level. The above results are consistent with the previous literature reports^14–16^. Most of the previous theoretical works took U = 4 eV^14–16^. However, it should be noted that in many calculations, the value of U has a great influence on the results. Therefore, we also conducted tests with different U values, where the U values range from 0 to 3 eV. The results of these calculations are presented in Figs. S2 and S3. In the FM state and the NM state, the band structures and flat band width change little with the U value. The width of the flat bands in all cases is summarized in Fig. S8a. The width of these flat bands of LK-99-2 is roughly between 50 and 160 meV. Some regularities can also be drawn from these results without SOC. First, the width of flat band2 is narrower than flat band1 in all cases. Second, spin polarization will reduce the width of the flat bands. Third, as the U value increases, the width of the flat bands will also increase slightly. In general, if the SOC is not considered, the flat bands in LK-99-2 are very robust, and the conclusions are not affected by the value of Hubbard interaction U.

### Electronic structure with SOC

**Figure 3:**
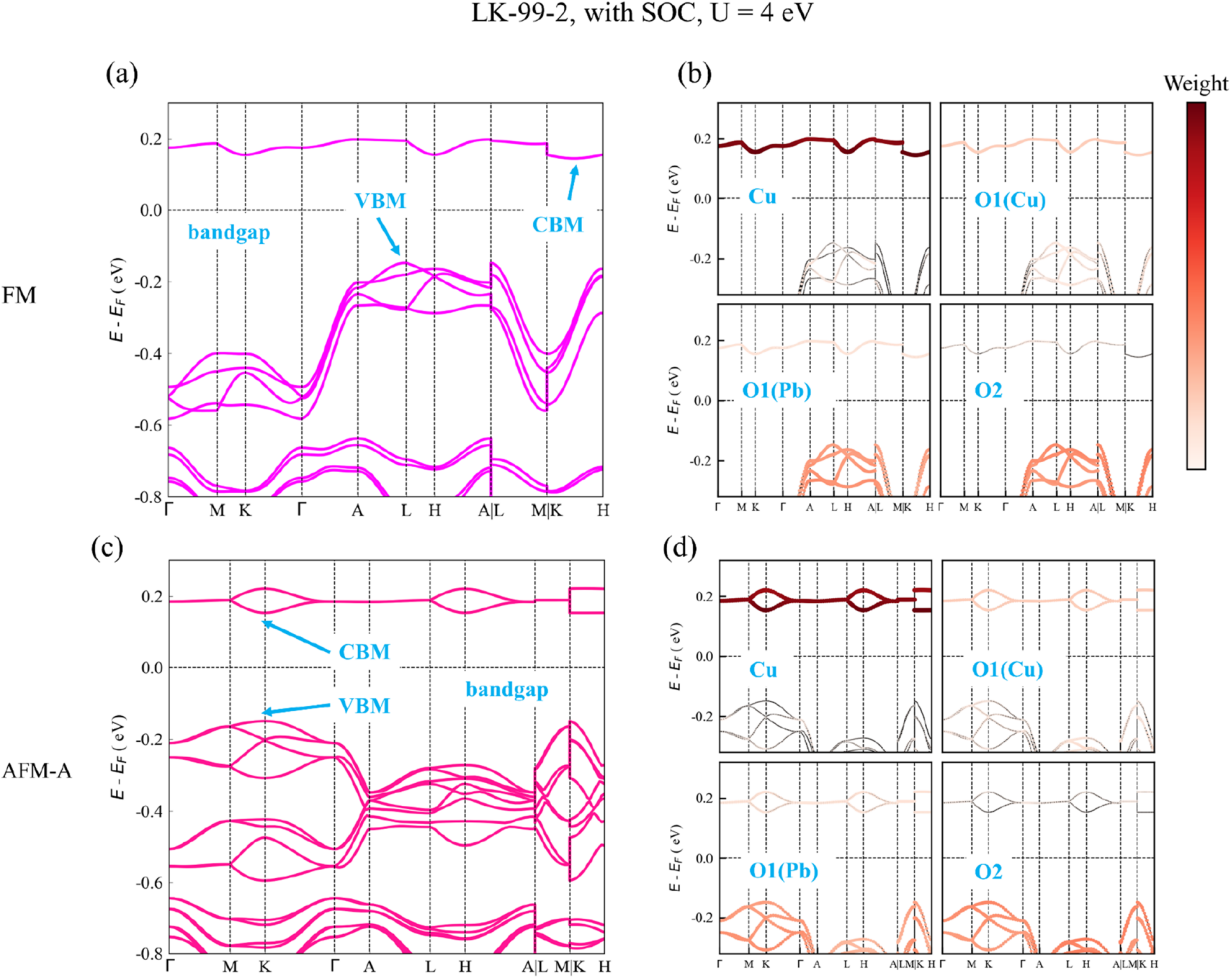
(**a**,**c**) Band structures of LK-99-2 with SOC and the Hubbard interaction U = 4 eV in FM state and AFM-A state, respectively. (**b**,**d**) Corresponding projected band structures around flat bands in FM state and AFM-A state, respectively.

The calculation results without considering the SOC look very interesting. But it should be noted that Pb is a heavy element with a relative atomic mass of 207.2, so the effect of SOC is often not negligible^24^. It is necessary to consider the electronic structure calculation with SOC. The direction of the magnetic moment is very important in calculations of the FM state with SOC, so the magnetic anisotropy energy (MAE) is calculated first. The MAE is defined as E_hard_–E_easy_, while E_hard_ and E_easy_ are the total energy of the system when the magnetic moment is parallel to the hard-axis and easy-axis, respectively. Calculations prove that the easy-axis of LK-99-2 is *c*-axis while hard-axis in the *ab*-plane and perpendicular to *b*-axis, and the MAE is calculated as 1.03 meV. Cu atoms contribute most of the magnetic moment. The three nearest neighbor O1 atoms around the Cu atoms also contribute a small amount of magnetic moment. The total magnetic moment is about 1 μB/unit cell. Other common antiferromagnetic (AFM) configurations are also considered, as shown in Fig. S4. The total energy difference of all magnetic configurations is very small, which may be due to the relatively large distance between Cu atoms and the relatively weak superexchange interaction. Considering that many experimental works have reported the FM state of LK-99^6,11–13^, but no AFM state, we first calculated the electronic structure of the FM state. Figure 3a shows the band structure of LK-99-2 with SOC and the Hubbard interaction U = 4 eV in the FM state. Unlike previous calculations which ignored the SOC, the band structure shows that LK-99-2 is an indirect-bandgap semiconductor with a bandgap of 292 meV. The conduction band minimum (CBM) and the valence band maximum (VBM) are located at the middle of K-H line and L point respectively. It should be noted that the conduction band is also a flat band, with a very narrow width of 52 meV. The corresponding projected band structures are shown in Fig. 3b. This flat band is mainly contributed by Cu atoms, while O1 atoms also have a small amount of contribution. The highest energy point and lowest energy point of this flat band are at A point and the middle of K-H line, respectively. In addition, the shape of this flat band is very similar to the shape of flat band2 in the previous calculation without SOC. Therefore, it can be judged that this flat band is the previous flat band2. Similarly, different values of U from 0 to 3 eV were also tested as shown in Fig. S5. The results show that, after considering the SOC, the value of U has a great influence on the band structure. When U = 0 eV, the band structure is very similar to the case without SOC. The situation gradually changes as the U value increases. When U = 1 eV, the position of flat band2 was raised, while the position of flat band1 was lowered, which eventually caused the band inversion of the two flat bands. At the same time, a bandgap of 0.114 eV is opened. As the U value increases, the value of the bandgap also increases, and the width of flat band2 decreases slightly. The bandgap of LK-99-2 under different conditions are summarized in Fig. S8b. When U greater or equal to 2 eV, flat band1 no longer exists in the energy range given in Fig. S5, and only one flat band is left around Fermi level. Since both flat bands are mainly contributed by Cu atoms, we projected the contribution of Cu on the band structures of LK-99-2 from − 5 to 5 eV when U = 4 eV in FM state without and with SOC, respectively, as shown in Fig. S6a and b. After considering the SOC, flat band1 move down to about – 4 eV. In addition, some states of Cu also move down from about − 2 eV to around − 4 eV. In short, the electronic structure of Cu has changed greatly after considering SOC. We speculate that there are two reasons: first, the neighbor effect of the SOC of the Pb element in this compound has a great impact on Cu; second, the SOC of the Cu element cannot be ignored here. In fact, it is reported that the SOC of Cu causes the band splitting of Cu-graphane at the Fermi level^25^. Another work also reported the effect of SOC of Cu adatoms on graphene^26^. However, the reason why SOC has such a big impact is still not very clear. This may be an issue worth studying in future works.

Next, the electronic structure of the AFM state with the lowest total energy, that is, the AFM-A state, is also calculated. Figure 3c and d shows the band structure and corresponding projected band structures of LK-99-2 in the AFM-A state. In this state, LK-99-2 is a direct-bandgap semiconductor with a bandgap of about 300 meV. Its CBM and VBM are both at K point. Its conduction band is composed of two flat bands, and its composition is basically the same as the flat band of the FM state, as Fig. 3d shows. In the experiments, the AFM state has not been observed. This may be because the energy difference between the AFM state and the FM state is too small, and the superexchange effect is too weak, so that the direction of the magnetic moment is easily reversed by the magnetic field. Finally, the ferromagnetic state is obtained in the magnetic field. We also consider the NM state with SOC. Although we try to fix the magnetic moment to 0 in VASP, the final solution still has some magnetic moments, and a strictly NM state is not obtained. Next, the electronic structure and corresponding projected band structures of LK-99-1 with SOC and U = 4 eV were also calculated, as shown in Fig. S7a and b. Like LK-99-2, after considering SOC, LK-99-1 is also an indirect bandgap semiconductor with a bandgap of 190 meV in FM state.

## Discussion

**Figure 4:**
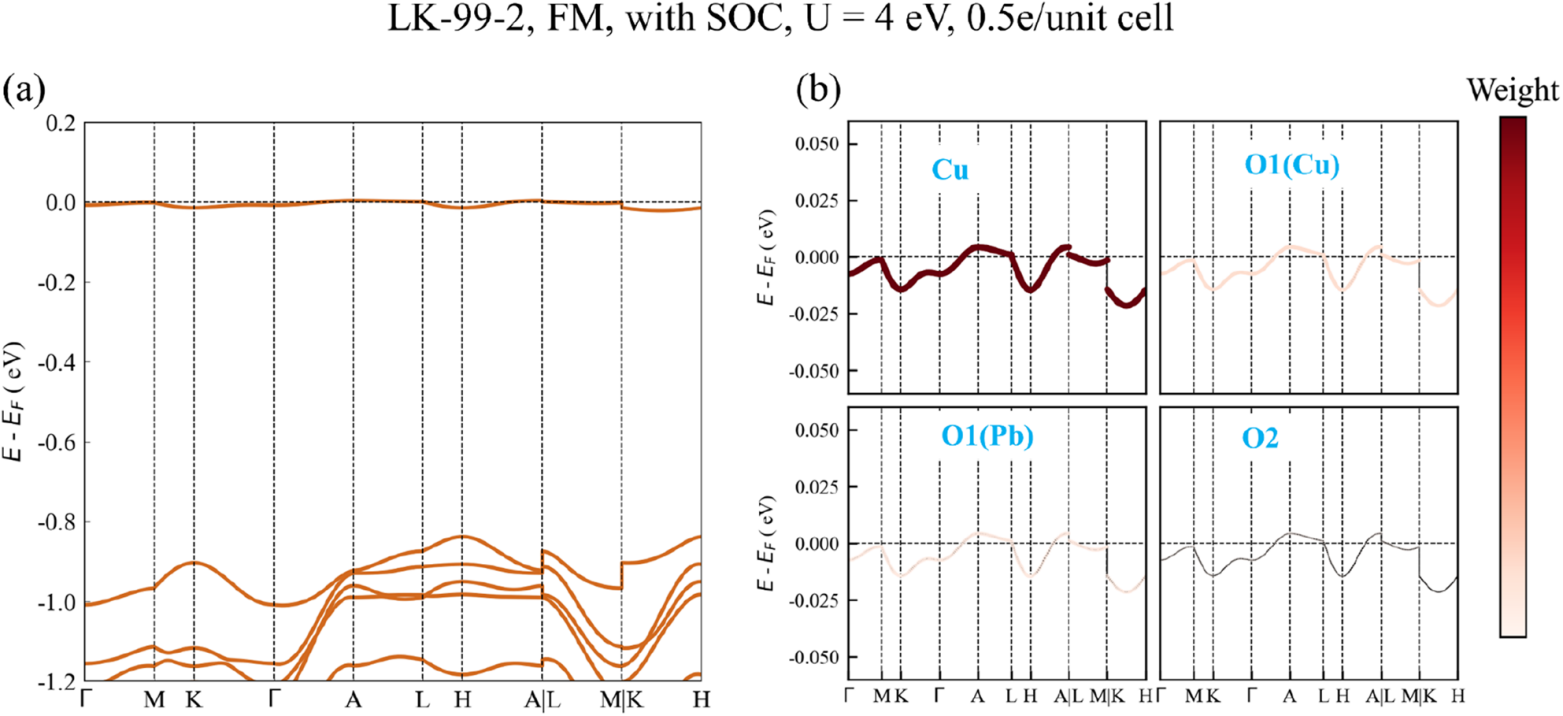
(**a**) Band structures of LK-99-2 with SOC and the doping with 0.5 electrons per unit cell in FM state. The Hubbard interaction U = 4 eV. (**b**) Corresponding projected band structures around flat band.

The flat bands near the Fermi level are considered to be one of the reasons for high-temperature superconductivity^27^, because these flat bands will cause a large density of states (DOS) near the Fermi level. According to the Bardeen-Cooper-Schrieffer (BCS) theory, this is beneficial to increase the *T*_C_ of superconductivity^28^. Flat bands are essentially the localization of electrons in real space. And there are several different mechanisms of flat band in different systems, such as heavy-fermion systems^29^, twisted two-dimensional systems^30^, kagome lattice systems^31^ and doped semiconductors or insulators^32^. If SOC is not considered, our calculation results and some previous calculation works are more in line with this situation, i.e. there are flat bands at the Fermi level. And this flat band is more likely to be caused by doping in the insulator, because the largest contribution to this flat band is Cu, that is, most of the states near the Fermi level are localized on Cu atoms. But as mentioned above, in a system with 9 Pb atoms in a unit cell, ignoring the SOC is imprecise and the results are often unreliable. In fact, some experiments trying to reproduce LK-99, the result of the resistance measurement is a semiconductor^6,9–11^, which is not consistent with the calculated metallic behavior. In addition, as a flat band system, LK-99-2 should have a relatively strong electron correlation, so we are more inclined to the calculation results with a relatively large U value, i.e. LK-99-2 is a semiconductor both in the FM state and in the AFM-A state. As a magnetic narrowband semiconductor, LK-99-2 also has some possible potential applications. For example, optoelectronic device^33^, photocatalytic^34^, photodetector^35^, spintronics device^36^. Additionally, in order to get the flat band on the Fermi level, we can make some modulations to LK-99-2. For example, a small amount of electronic doping allows the conduction band to be partially occupied, and a flat band at the Fermi level can be obtained. Therefore, we tried to calculate the electronic doping of LK-99-2 after considering the SOC in the FM state. As shown in Fig. 4a, after doping 0.5 electron per unit cell, the flat band becomes partially occupied, and the width of this flat band is only 25 meV. The corresponding projected band structures in Fig. 4b show that this flat band is mainly contributed by Cu atoms, and O1 atoms also have a small amount of contribution.

It should be noted that our calculations are only focus on two simple structures (LK-99-1 and LK-99-2) with the chemical formula of Pb_9_Cu_1_(PO_4_)_6_O, which are the second and most stable structures considering only x = 1 with the Cu atoms occupying the Pb1 sites. These are just some of the possible structures of LK-99. If our proposed structures are experimentally successfully synthesized, they should be semiconducting due to the SOC. Depending on the number and position of Cu atoms and O2 atoms, LK-99 has other more complex structures with larger unit cells. In addition, it is impossible to judge whether a compound is a superconductor or not only from the calculations of the electronic structure. Even if there is a flat band at the Fermi level, it does not mean that it is a superconductor.

## Conclusions

In conclusion, our first-principles calculations show that SOC is non-negligible in Pb_9_Cu(PO_4_)_6_O. If the SOC is not considered, although the calculation can get the flat bands on the Fermi level, this result is not rigorous and does not agree with some experiments. Our calculations show that once the SOC is considered, a bandgap is opened, making Pb_9_Cu(PO_4_)_6_O an indirect-bandgap semiconductor with a bandgap of 292 meV and a flat conduction band in the FM state. After electron doping at the level of 0.5 e/unit cell, it becomes metallic and has a flat band with a width of only 25 meV at the Fermi level in the FM state. While in AFM-A state, Pb_9_Cu(PO_4_)_6_O is a direct-bandgap semiconductor with a bandgap of 300 meV. Our calculation results provide a possible explanation for the inconsistencies of some previous experimental and theoretical works^6,9–11^, make up for the lack of previous theoretical works^14–20^, point out the importance of considering SOC in the theoretical work of LK-99, and also provide some suggestions for experimental works. In addition, our work also provides some theoretical basis for the possible potential application of Pb_9_Cu(PO_4_)_6_O as a magnetic narrowband semiconductor.

## Methods

In this work, the calculations were performed using the Vienna ab initio simulation package (VASP)^37^ with the projector-augmented wave (PAW) method^38^. The generalized gradient approximation (GGA) with Perdew, Burke, and Ernzerhof (PBE)^39^ realization was used for the exchange–correlation function. The cutoff energy was set above 450 eV. The force and energy convergence criteria were set to 0.01 eV/Å and 10^−5^ eV, respectively. A 5 × 5 × 7 Γ-centered *k*-point mesh was used for the Brillouin zone sampling. In order to better compare with the experimental results, in the process of structure optimization, we fixed the lattice constants as the experimental value: *a* = 9.843 Å and *c* = 7.428 Å^1^. The electronic correlation effects of Cu 3d orbits were described using the DFT + U method^40^, and the values of U are carefully tested from 0 to 4 eV. The impact of different values of U on the structure has also been considered, so the structures of all systems under different values of U have been fully optimized.

### Supplementary Information

Supplementary Figures.

## Data Availability

The data that support the findings of this study are available from the corresponding author upon reasonable request.
