# Supplementary material for: Semiconductivity induced by spin–orbit coupling in Pb9Cu(PO4)6O
Source: Sci Rep. 2023 Nov 30;13:21085. doi: 10.1038/s41598-023-48383-z (PMC10686976; doi:10.1038/s41598-023-48383-z)
Supplement: Supplementary file 1 — Supplementary Figures. [file 41598_2023_48383_MOESM1_ESM.pdf]

Supplementary Information

Semiconductivity induced by Spin-orbit Coupling in $Pb_9Cu_1(PO_4)_6O$

Hua Bai[1]*, Jianrong Ye[1], Lei Gao[1], Chunhua Zeng[1]*, Wuming Liu[2]*

[1]*Institute of Physical and Engineering Science/Faculty of Science, Kunming University of Science and Technology, Kunming 650500, China*
[2]*Beijing National Laboratory for Condensed Matter Physics, Institute of Physics, Chinese Academy of Sciences, Beijing 100190, China*
*Corresponding authors: huabai@kust.edu.cn (H.Bai); chzeng83@kust.edu.cn (C.Zeng); wliu@iphy.ac.cn (W.Liu)

CONTENTS

S1. Band structures and of LK-99-1 with out (w/o) Spin-orbit Coupling (SOC) with U = 4 eV **2**
S2. Band structures of LK-99-2 w/o SOC with different U **3**
S3. Schematic diagrams of different magnetic configurations **5**
S4. Band structures of LK-99-2 with SOC with different U **6**
S5. Projected band structures of Cu of LK-99-2 w/o and with SOC **7**
S6. Band structures of LK-99-1 with SOC with U = 4 eV **8**
S7. The summary of flat band width and bandgap of LK-99-2 **9**

S1. Band structures and of LK-99-1 with out (w/o) Spin-orbit Coupling (SOC) with U = 4 eV

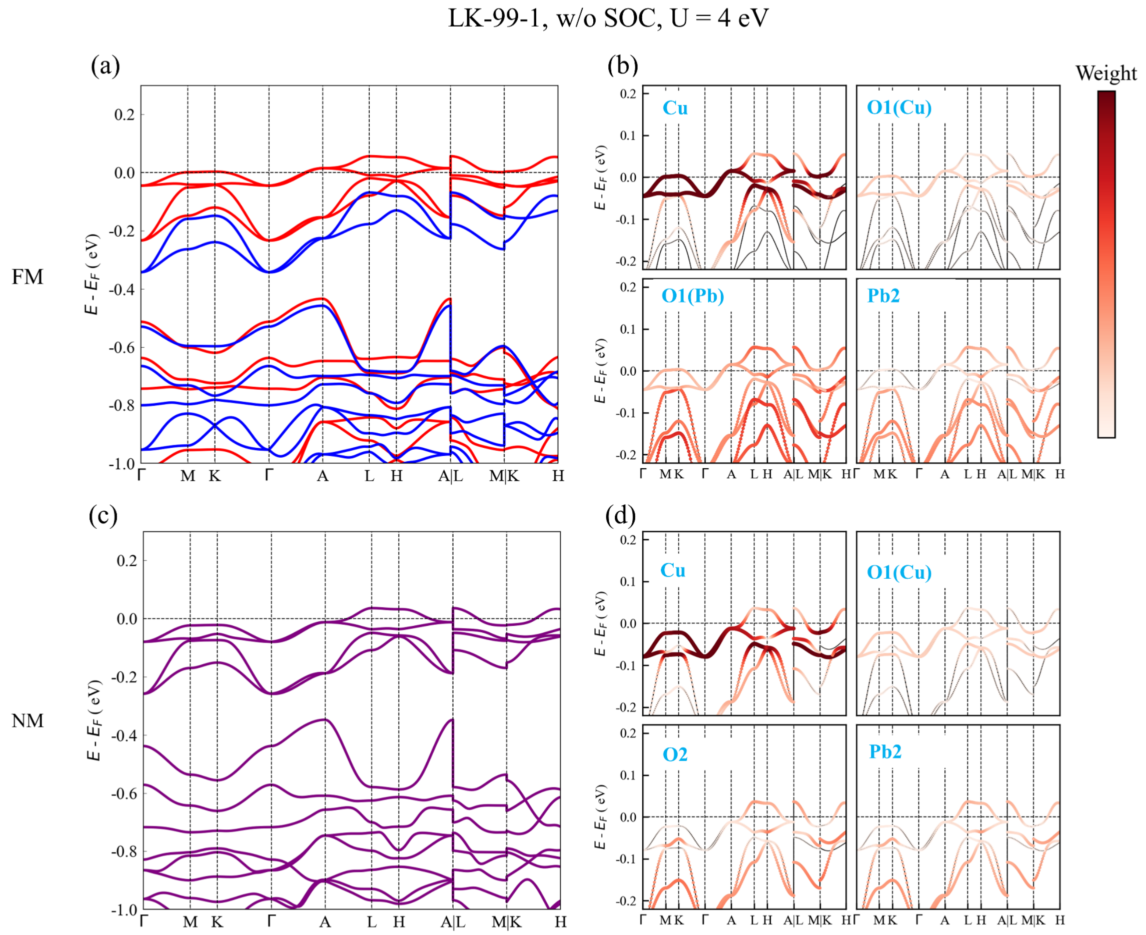

Figure S1. (a) (c) Band structures of LK-99-1 without SOC (w/o SOC), and the Hubbard interaction U = 4 eV in FM state and NM state, respectively. (b) (d) Corresponding projected band structures around flat bands in FM state and NM state,

S2. Band structures of LK-99-2 w/o SOC with different U

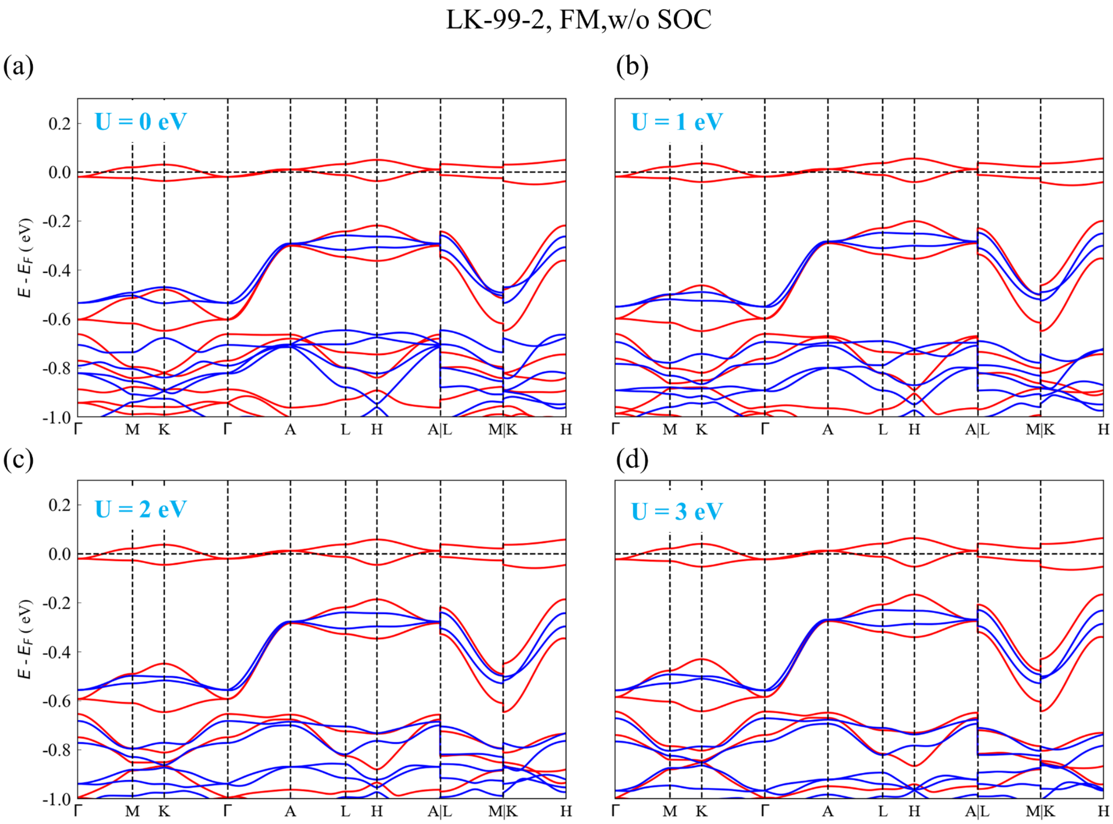

Figure S2. Band structures of LK-99-2 without SOC, and the different Hubbard interaction U from 0 to 3 eV in the FM state.

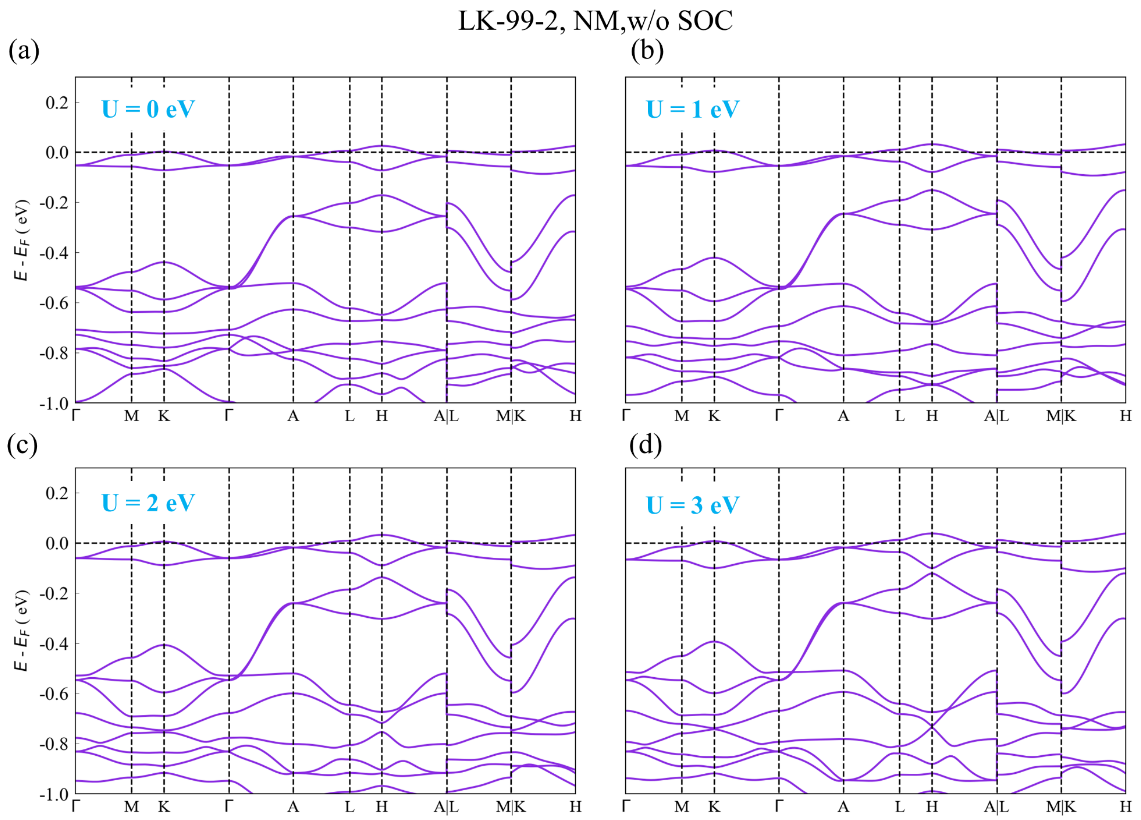

Figure S3. Band structures of LK-99-2 without SOC, and the different Hubbard interaction U from 0 to 3 eV in the NM state.

S3. Schematic diagrams of different magnetic configurations

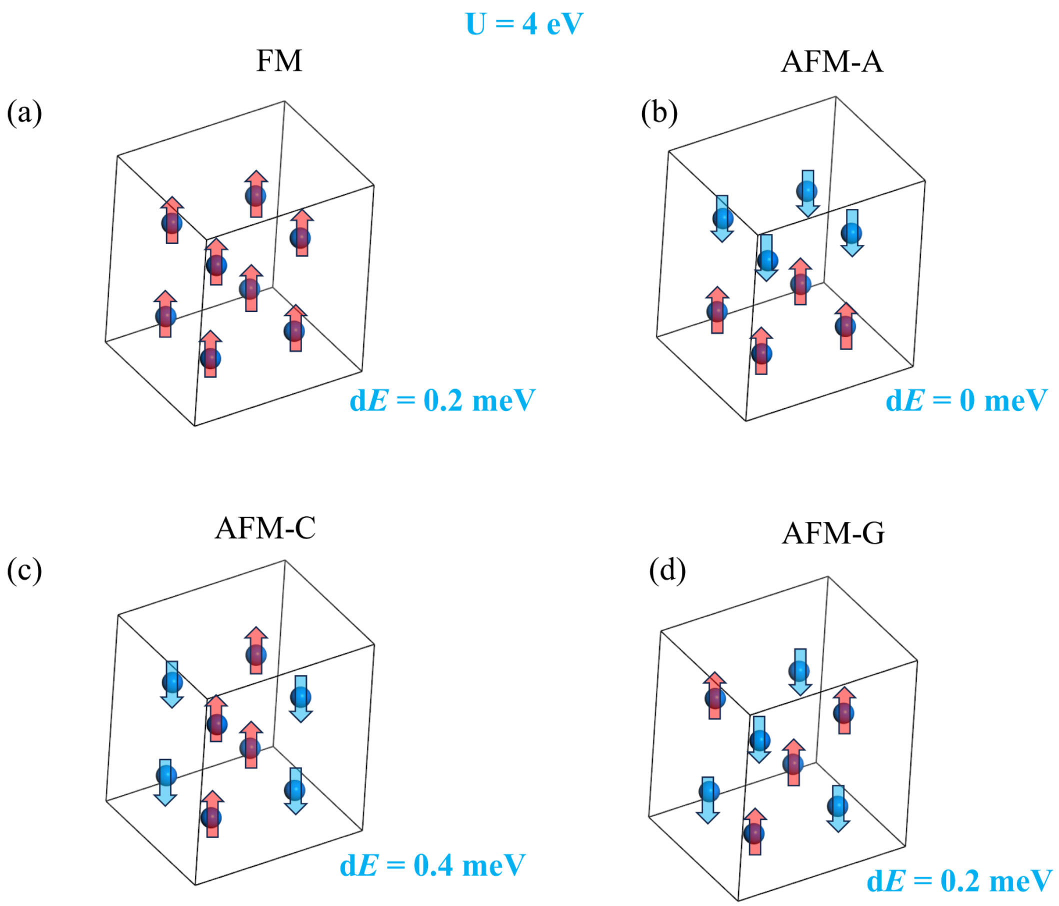

Figure S4. Schematic diagrams of different magnetic configurations in 2*2*2 supercell. For clarity, only Cu atoms are shown. Arrows indicate the direction of the magnetic moments. (a) Ferromagnetic (FM). (b) Antiferromagnetic-A (AFM-A). (c) AFM-C. (d) AFM-G. The blue numbers represent the relative total energys (d*E*) with Hubbard interaction U = 4 eV while the total energy of AFM-A is set to 0 meV.

S4. Band structures of LK-99-2 with SOC with different U

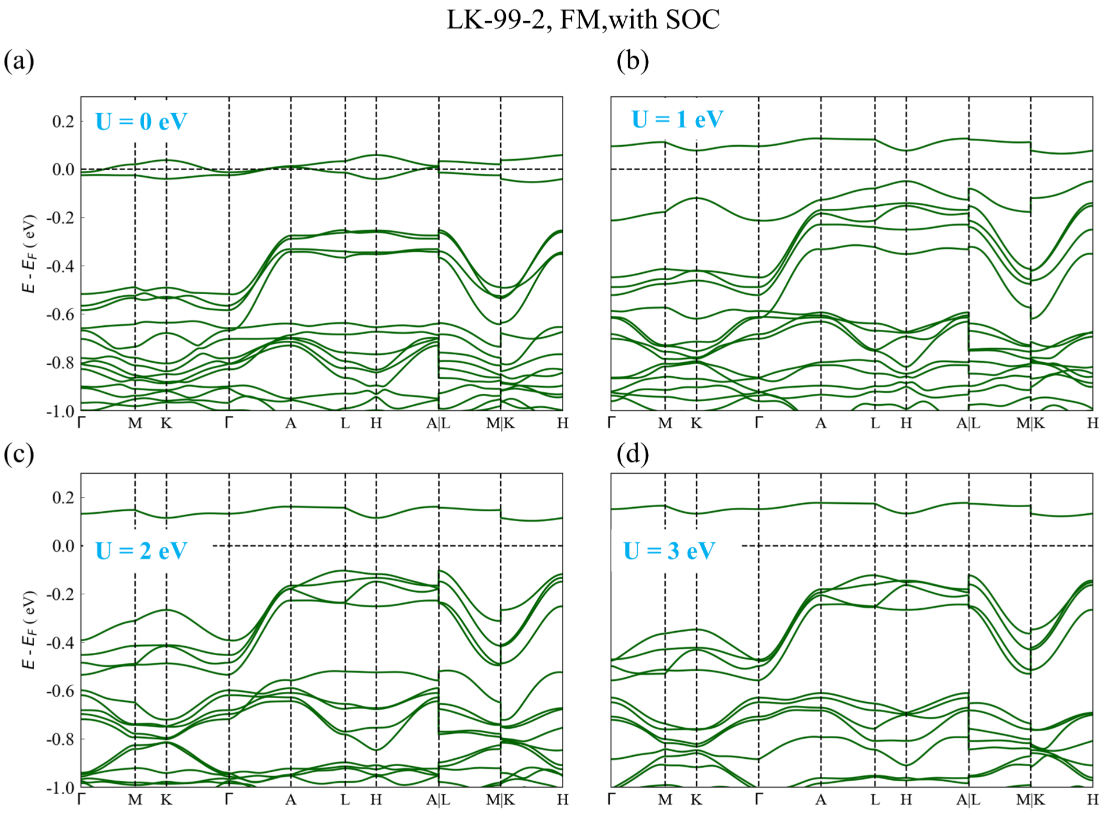

Figure S5. Band structures of LK-99-2 with SOC and the different Hubbard interaction U from 0 to 3 eV in the FM state. When U = 0 eV, the system is metallic. As U increases, the system becomes semiconducting and the bandgap increases.

S5. Projected band structures of Cu of LK-99-2 w/o and with SOC

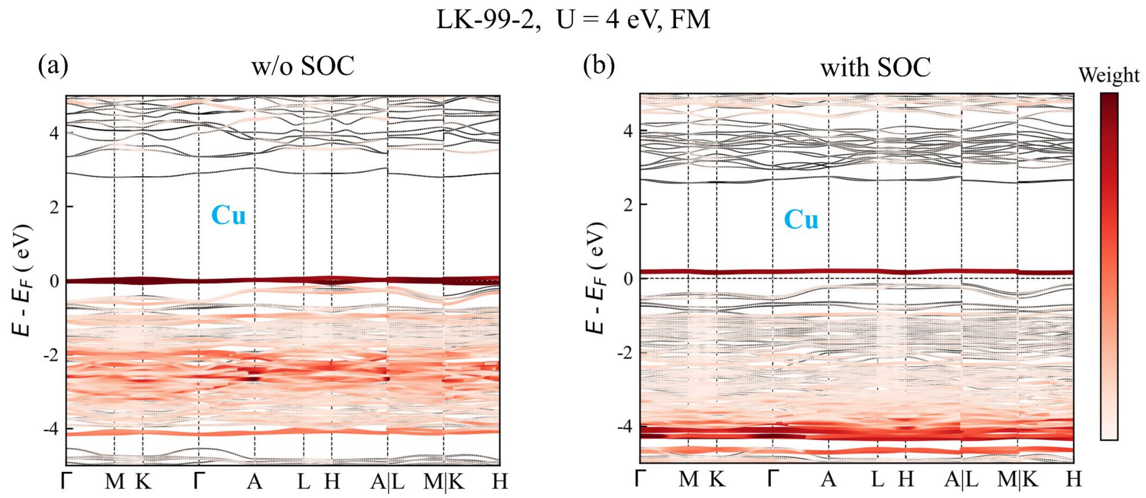

Figure S6. (a) (b) Projected band structures of Cu of LK-99-2 from -5 to 5 eV w/o and with SOC, respectively.

S6. Band structures of LK-99-1 with SOC with U = 4 eV

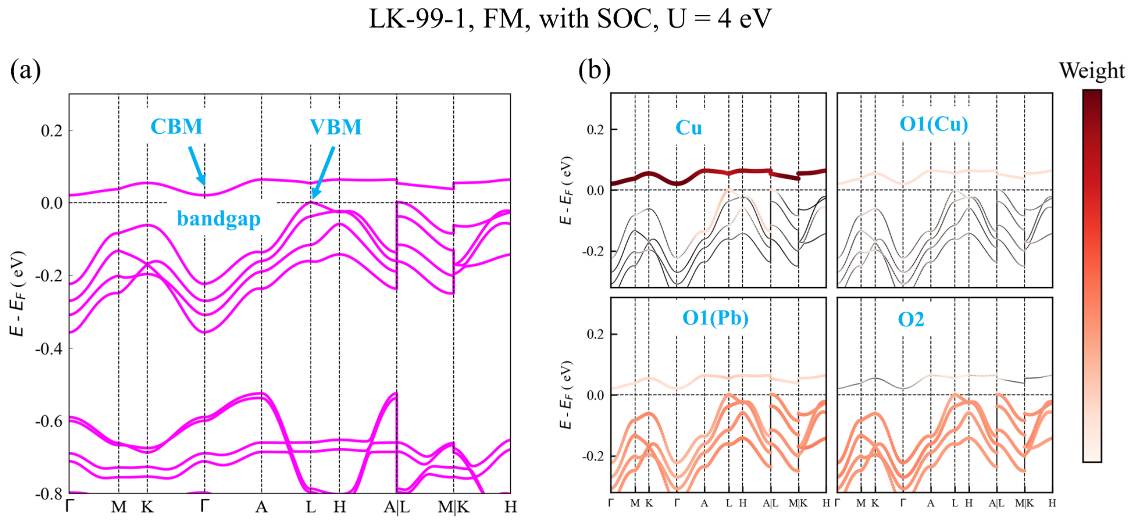

Figure S7. (a) Band structures of LK-99-1 with SOC and the Hubbard interaction U = 4 eV in FM state. (b) Corresponding projected band structures around flat bands.

S7. The summary of flat band width and bandgap of LK-99-2

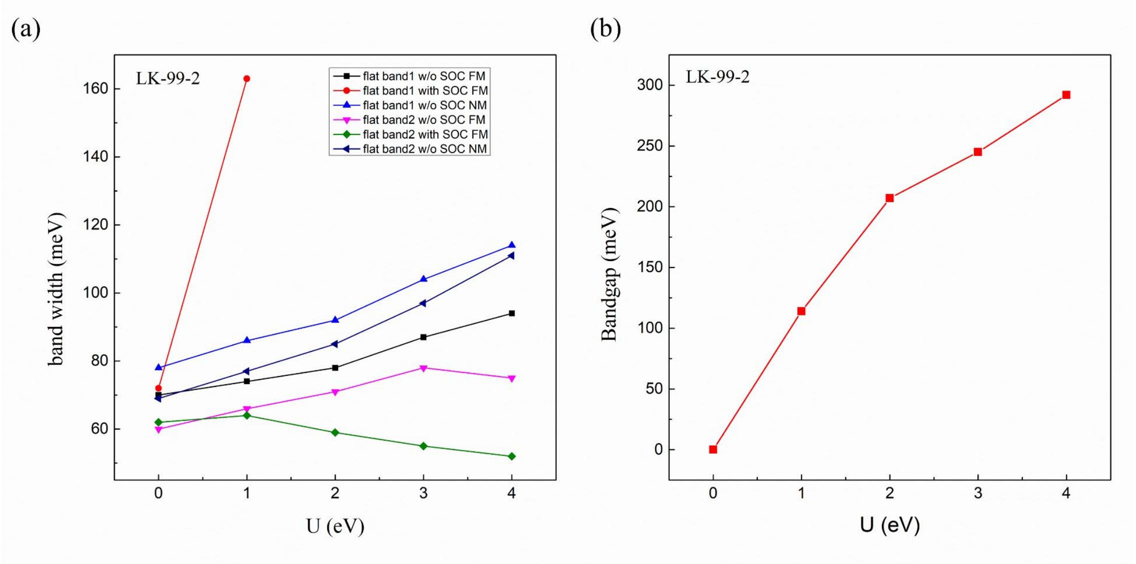

Figure S8. (a) The width of flat band1 and flat band2 of LK-99-2 with different Hubbard interaction U under different conditions (w/o SOC in NM state, w/o SOC in FM state, with SOC in FM state). FM and NM represent the ferromagnetic and non-magnetic states, respectively. (b) The summary of bandgap of LK-99-2 under different U from 0 to 4 eV in FM state. The bandgap increases with the increased value of U.
